# Supplementary material for: Chromothripsis during telomere crisis is independent of NHEJ, and consistent with a replicative origin
Source: Genome Res. 2019 May;29(5):737–49. doi: 10.1101/gr.240705.118 (PMC6499312; doi:10.1101/gr.240705.118)
Supplement: Supplemental Material [file supp_gr.240705.118_Supplemental_file_1.zip › contigs/annotated_contigs/DB114/contig.2.DB114_length_743_mean_cov_10.9448183042.docx]

**DB114_length_743_mean_cov_10.9448183042**

```
AACTTTCTTTTATAGAACCACAGGGAGAAATTGCAAAAGGGAAATTAAACAGAATTAAAAGGTAAACTAGAAAAAGACATTAACAATAT
 >chr12:80031866-80032267 - E=1e-224 p=2e-03
GTTGGACAAAGATTACCATTCTTGTTATATACTGCTTTTAGAAGTACTAAGAAATGAAATTGACAGACAACATCAGTGAAGAAAATGAA

GATACATAAATATATAAAGCCAATAAAAATATGACAAAAGTTTATCCTATTAATTAGGATTCAGTGAAACCCCTATTTGCCTAATATAT

TTGGTGAGAGTTGAAAAAATAAGGGTGAGACCAATCTTACACAAGCTTTCTGGGGCAATATTGGCAGTATTTTTTTTTTTTGAAACCCA

AAAGCTGTACTCTGAAGAATGACAGTATGTTTAAAGAGCC|ATAA|GAGAAGCAAGTGTATGTGGGGGGATATTAAGTTCCATTTCATG
                                         >chr12:80338178-80338474 - E=3e-166
TATGTTAGGCTTGAAGATTTCATGTCAGTAGGCAGTCGTGTAAATAGGTATGGGACTTAGGTGCAATATTTTGCCTTGAGGTGGGGATC

TGAGAAAAATTAAATAGAAAAAAGAGCTAAAGATGAAATCCTGTGAAATAAGATGTACGCAAAGAAAAAGGGGGCTTATAATAGGAGTC

TGAAAAAATTAGAGAGTTAGGAAAACCCAGAAGAAAGTAGTGTCATGGAAGACAAGGCGAAAATTTCCAGA|AGATCGGAAGAGCACAC

GTCTGAACTCCAGTCACTCCGGAGAATCCCGTAT
```
